# Improving maternal and child health policymaking processes in Nigeria: an assessment of policymakers’ needs, barriers and facilitators of evidence-informed policymaking

**DOI:** 10.1186/s12961-017-0217-5

**Published:** 2017-07-12

**Authors:** Chigozie J. Uneke, Issiaka Sombie, Namoudou Keita, Virgil Lokossou, Ermel Johnson, Pierre Ongolo-Zogo

**Affiliations:** 1 Knowledge Translation Platform, African Institute for Health Policy & Health Systems, Ebonyi State University, PMB 053 Abakaliki, Nigeria; 2 West African Health Organisation, 175 avenue Ouezzin Coulibaly, 01 BP 153 Bobo-Dioulasso 01, Burkina Faso; 3 Hopital Central Yaounde, CDBPH Lawrence VERGNE Building 2nd Floor, Avenue Henry Dunant Messa, Yaoundé, Cameroon

## Abstract

**Background:**

In Nigeria, interest in the evidence-to-policy process is gaining momentum among policymakers involved in maternal, newborn and child health (MNCH). However, numerous gaps exist among policymakers on use of research evidence in policymaking. The objective of this study was to assess the perception of MNCH policymakers regarding their needs and the barriers and facilitators to use of research evidence in policymaking in Nigeria.

**Methods:**

The study design was a cross-sectional assessment of perceptions undertaken during a national MNCH stakeholders’ engagement event convened in Abuja, Nigeria. A questionnaire designed to assess participants’ perceptions was administered in person. Group consultations were also held, which centred on policymakers’ evidence-to-policy needs to enhance the use of evidence in policymaking.

**Results:**

A total of 40 participants completed the questionnaire and participated in the group consultations. According to the respondents, the main barriers to evidence use in MNCH policymaking include inadequate capacity of organisations to conduct policy-relevant research; inadequate budgetary allocation for policy-relevant research; policymakers’ indifference to research evidence; poor dissemination of research evidence to policymakers; and lack of interaction fora between researchers and policymakers. The main facilitators of use of research evidence for policymaking in MNCH, as perceived by the respondents, include capacity building for policymakers on use of research evidence in policy formulation; appropriate dissemination of research findings to relevant stakeholders; involving policymakers in research design and execution; and allowing policymakers’ needs to drive research. The main ways identified to promote policymakers’ use of evidence for policymaking included improving policymakers’ skills in information and communication technology, data use, analysis, communication and advocacy.

**Conclusion:**

To improve the use of research evidence in policymaking in Nigeria, there is a need to establish mechanisms that will facilitate the movement from evidence to policy and address the needs identified by policymakers. It is also imperative to improve organisational initiatives that facilitate use of research evidence for policymaking.

**Electronic supplementary material:**

The online version of this article (doi:10.1186/s12961-017-0217-5) contains supplementary material, which is available to authorized users.

## Background

Within the last decade, there has been unprecedented global interest in the promotion of the use of research evidence to inform policymaking in the health sector. This interest was triggered by the 2005 World Health Assembly Resolution, which strongly encouraged member states to harness health research more effectively to achieve the United Nations Millennium Development Goals (MDGs), and especially in low- and middle-income countries (LMICs) [1].

The use of research evidence in policymaking is no longer limited to high-income countries, and evidence-informed policy is growing in importance among policymakers in LMICs [2–5]. A previous report quoted the Director of the Tanzanian Council for Science and Technology as saying, “*if you are poor, actually you need more evidence before you invest, rather than if you are rich*” [6]. International meetings in Mexico City in 2004 and later in Bamako, Mali, in 2008, also emphasised the importance of promoting the conduct and use of essential health systems research, securing public confidence in research and bridging the gap between knowledge and action in developing countries [7, 8].

Despite the worldwide recognition of the dire need for health policy to be informed by research evidence, several reports have unequivocally proven that health policies, especially in most LMICs, are not well-informed by research evidence [9, 10]. For Holmes et al. [11], the gap between the evidence generated through research and that which is applied in healthcare is becoming too large to ignore. Oxman et al. [12] have argued that poorly-informed decision-making is one of the reasons why services sometimes fail to reach those most in need, why health indicators became off-track, and why many LMICs were unable to meet the health MDGs.

Nigeria is among the LMICs that were unable to attain the health MDGs. With a current population of over 160 million, WHO ranked the country’s health systems at 187th out of 191 member states in 2000 [13]. Since then, health outcomes, especially those related to MNCH, have remained suboptimal. Available reports indicate that Nigeria has more than 10% of all under-five and maternal deaths – more than one million newborn, infant and child deaths, and more than 50,000 maternal deaths every year [14–16].

Nonetheless, there has been some improvement within the last decade in MNCH outcomes in Nigeria. The national maternal mortality ratio has reportedly reduced from 800/100,000 in 2005 [16, 17] to 576/100,000 in 2013 [18]. Further, the under-five mortality rate in Nigeria reduced from 201 per 1000 live births in 2003 [19, 20] to 117 per 1000 live births in 2013 [21]. This improvement in MNCH outcomes in Nigeria may be in part attributed to the increasing awareness of the importance of evidence-informed policymaking among decision-makers [22–24], and the implementation of various MNCH policies, some of which are clearly based on research evidence [25–28]. Other factors, such as improvement in health systems, health sector leadership/governance and increased health financing, have contributed to the reduction in the national maternal mortality ratio within the last decade [18].

The process of getting research evidence into policy and practice, i.e. bridging the know-do-gap, is not a simple venture and, in Nigeria, the process is well recognised as a complex one [22, 29]. This is because the process of evidence-informed policymaking is characterised by multiple barriers and facilitators that are country and context specific. Commonly identified barriers include lack of policy relevant research, lack of political support, weak administrative structure for policymaking, lack of trained policymakers in accessing and using evidence, as well as low demand for scientific evidence by policymakers [30–32]. Among the main facilitators are improved funding for policy-relevant research, easy access to policy relevant research findings, communication and networking between policymakers and researchers, wide dissemination of research, and enhancing policymakers’ skills for evidence-informed policymaking [30, 32]. Several previous reports have clearly indicated that the successful implementation of strategies to address the know-do-gap is highly dependent on the identification of the barriers and facilitators of uptake of research into policy in each specific setting [33–35].

There is an urgent need for the design and execution of specific intervention strategies and programmes that will address the evidence-to-policy barriers in the Nigerian context. According to WHO [36], to ensure implementation of an evidence–response mechanism that is targeted and effective, a thorough assessment of needs is required, and such an assessment should consider the evidence needs and relative priorities of stakeholders within and across countries as well as a systematic assessment of the evidence gaps around maternal and child health in the region.

Although the awareness and interest in evidence-informed policymaking has gained momentum in Nigeria, meeting points, such as policymakers’ engagement events to consider issues around the research–policy interface related to MNCH, are essentially lacking. It is pertinent to state that, in addition to research, evidence for policymaking can be derived from knowledge and information, ideas and interest, and the wider political and economic environment [37]. Bowen and Zwi [38] have noted that evidence encompasses research, and may include opinions and views of individuals or groups, results of consultative processes, and published reports and documents. However, evidence from scientific research has been consistently shown to be among the most reliable categories of evidence in the development and implementation of health policy capable of producing better health outcomes [39, 40]. Our focus in this study was on the use of research evidence in policymaking. The objective of this study was to assess the perception of MNCH policymakers regarding their needs and the barriers and facilitators to use of research evidence in policymaking in Nigeria. This was as part of the effort to promote evidence-to-policy-to-practice processes for the improvement of MNCH outcomes in Nigeria.

## Methods

### Study design and participants

The study design is a cross-sectional assessment of the perception of policymakers and other key stakeholders regarding their needs and the barriers and facilitators to use of research evidence in policymaking in Nigeria. The study was conducted among 40 participants at a one-day national MNCH stakeholders’ engagement event convened under the auspices of the West African Health Organisation (WAHO) and the Nigerian Federal Ministry of Health (FMOH), in October 2015 in Abuja, Nigeria. The participants were senior staff of various organisations involved in the policymaking process and included the FMOH Abuja and its associated ministries, departments and agencies, the state ministries of health, development partners, civil society organisations, non-governmental organisations and universities/research institutes. The purpose of the meeting was to promote the use of research evidence in policymaking and practice regarding MNCH. We were not funded to do formal research, as the funding obtained from WAHO was to convene a national stakeholder dialogue. We therefore designed the event to explore whether and how information could be obtained that could be considered quality evidence to inform future capacity building efforts. This represents an example of applying learning-by-doing strategies and using available processes to more systematically identify priorities and issues.

### Data collection tool and technique

#### Self-assessment questionnaire

The use of a self-assessment questionnaire was employed in this study, and the questionnaire is provided in Additional file 1. The questionnaire was given to the 40 participants during the meeting, all of whom completed and returned it. In the development of the questionnaire, we drew insights from the self-assessment tool produced by the Canadian Health Services Research Foundation [41]. This self-assessment tool was consulted based on available reports that indicated its usefulness in the assessment of individual and organisational capacity in the use of research evidence in the design and delivery of services [24–28]. The questionnaire was designed as an open-ended instrument that centred on the following within each participant’s organisation: (1) existing mechanisms, processes, tools, strategies and platforms for research evidence use in policymaking; (2) existing monitoring, evaluation and performance assessment mechanisms for research evidence use in policymaking; (3) factors limiting research evidence use in policymaking; and (4) possible strategies to address the limiting factors for research evidence use in policymaking.

#### Group consultation and group presentations

Group consultations were held in which the participants were grouped according to their organisation type. The group consultation was centred on policymakers’ evidence-to-policy needs to enhance the use of evidence in policymaking and lasted up to 70 minutes.

A total of five organisational group consultations were conducted as follows:

- Group 1: Participants from the FMOH.
- Group 2: Participants from ministries, departments and agencies.
- Group 3: Participants from state ministries of health.
- Group 4: Participants from development partners, non-governmental organisations, civil society organisations.
- Group 5: Participants from professional associations and research institutions.

Each group had between 7 and 12 participants, and each was led by a participant who was a senior staff of their organisation selected by other participants in the group based on their previous experience leading such discussions. The leader of each group was provided with a guideline they used to facilitate the consultation. The group consultations were not recorded, but each group appointed a participant to take notes on the key issues identified. Eight key topical issues related to capacity for evidence use in policymaking were discussed by each group and comments and resolutions were documented. The topical issues were categorised as individual capacity or organisational capacity. Each group was requested to articulate and summarise the key issues identified and discussed into bullet points and short phases. A representative from each group made a presentation on the outcome of group consultations during plenary. The list of questions used is as follows:

- Individual capacity
  1. *Aptitudes*: To strengthen your aptitudes towards the use of research evidence, what are the interventions that are important to you?
  2. *Skills*: What types of skills do you need to better use research evidence and research findings?
  3. *Sources of evidence*: Which sources of evidence or research results would you like to access to improve your use of research evidence and research findings?
  4. *Formats of evidence*: In what formats would you like to receive research evidence and research findings to help you use them?
- Organisational capacity
  1. *Institutional environment*: What types of improvements (laws, regulations, service organisation, support, motivation, etc.) do you think are important in your workplace that can help you to better use research evidence and research findings?
  2. *Platforms or mechanisms*:
    - What types of platforms or mechanisms do you think are important to put in place or reinforce in your workplace to facilitate your access to research evidence and research findings?
    - What types of platforms or mechanisms do you think are important to put in place or reinforce in your workplace to facilitate your everyday use of research evidence and research findings?
  3. *Opportunity of use of evidence*: What activities in your daily work are opportunities for you to engage in permanent use of research evidence and research findings?
  4. *Needs support*: What types of support would you like to receive when you decide to use research evidence and research findings?

Another round of general deliberation was undertaken and the key comments were articulated together. The plenary lasted up to 45 minutes.

### Analysis of participants’ response

The written responses from the questionnaires and the notes from the group consultations were analysed using Giorgi’s Phenomenological Approach [42], which was further elaborated by Albert et al. [43]. The analysis was conducted by (1) going over all the textual information; (2) identifying all comments that appeared significant; (3) abstracting the meaning units, (4) categorising and summarising abstractions; and (5) returning to the extracted text to ensure a good fit.

## Results

### Biodata and official designation attributes

Of the 92 participants, 71 (77.2%) were from organisations involved in policymaking processes in Nigeria (i.e. FMOH, ministries, departments and agencies, state ministries of health, development partners, non-governmental organisations). Of the 71 participants from these organisations, 40 (56.3%) signed the informed consent form and completed the policymakers’ questionnaire and participated in the group consultation. The profile of the 40 participants is presented in Table 1. A total of 24 (60.0%) of the respondents were female, and up to 64% of the respondents were more than 44 years old. The FMOH and its associated ministries, departments and agencies had the largest proportion of representatives (45%). Most of the respondents (59%) were either directors or chairpersons in their organisations. Most of the respondents had either spent 3–5 years (45%) or less than 3 years (37.5%) in their present designation. A total of 59% of the respondents reported that they have direct influence on policymaking processes. The results from the questionnaire and the group consultations were combined. Our decision to combine the results was informed by the fact that most of the participants’ responses were very similar. This suggested that, with respect to the evidence-to-policy process, individual and organisational capacity constraints are similar irrespective of organisational affiliation. Using Giorgi’s Phenomenological Approach [38], we focused more on the textual information and comments that appeared significant with regards to MNCH and the evidence-to-policy process.

**Table 1:** Profile of participants who completed the questionnaire and took part in the focus group discussions at the stakeholders’ engagement event

Parameter assessed	Outcomes (%)
Sex	
Male	16 (40.0)
Female	24 (60.0)
Total	40
Age category, years	
25–34	4 (11.1)
35–44	9 (25.0)
>44	23 (63.9)
Total	36
Type of organisation	
Federal Ministry of Health/ministries, departments and agencies	18 (45.0)
State ministries of health	10 (25.0)
Non-governmental/civil society organisations	5 (12.5)
Development partners	3 (7.5)
Others	4 (10.0)
Total	40
Designation	
Director	23 (59.0)
Manager/head of department	5 (12.8)
Programme/project officer	11 (28.2)
Total	39
Duration in designation, years	
<3	15 (37.5)
3–5	18 (45.0)
6–10	5 (12.5)
>10	2 (5.0)
Total	40
Influence on policymaking process	
Direct	23 (59.0)
Indirect	16 (41.0)
Total	39

Below are some of the key findings, organised by topical issue

### Individual capacity for use of research evidence

#### Aptitudes

In terms of strengthening aptitudes for use of evidence and the interventions that are important, the various groups emphasised the use of capacity building, creating enabling environments to support evidence, and provision of incentives/reward to encourage stakeholders to promote use of evidence (Table 2).

**Table 2:** Participants’ response on individual capacity needs for support of the use of evidence in policymaking relevant to maternal, newborn and child health

Aptitudes	Skills	Sources of evidence	Forms of evidence
(1) Capacity building such as training, internship to train younger officers, provision of necessary equipment to work with, etc. (2) Incentives such as reward, promotion (3) Networking through information sharing (4) Intersectoral and intrasectoral coordination (5) Create an enabling environment that supports the use of evidence in the workplace (6) Ability to generate high level evidence from randomised controlled trials, intervention research and systematic reviews (7) Ability to identify relevant policy areas/gaps for research	(1) Information communication technology skills (2) Information gathering, data analysis and interpretation (3) Communication skills-written and verbal (4) Scientific writing and communication (5) Research methodology (6) Advocacy skills (7) Individual capacity to know the types of evidence, sources of evidence and level of evidence (8) Capacity identify research evidence (extract, synthesise and present research evidence) (9) Skills of critical literature review, understanding basic statistics	(1) Publications –journals, surveys, books (2) Library – hard and soft copies (3) Internet/electronic resources for articles and journals (4) Dissemination forums for research findings (5) Development of local database of research	(1) Hardcopies – summaries/abstract and prose report (2) Newsletters and videos (3) Success stories and testimonials (4) Policy briefs/leaflets (5) Scientific meeting and publications from conference proceedings (6) Knowledge sharing and feedback mechanisms

#### Skills required

Participants emphasised their need for information and communication technology (ICT) skills, skills in research methodology and scientific writing, skills in data gathering, use, analysis and management, and skills in communication and advocacy (Table 2).

#### Sources of evidence

The need for availability and accessibility of publications, including peer-reviewed journals and other articles both in hard copy and electronically, was emphasised by participants. They also noted the need for the development of local databases of research and evidence, and dissemination forums for research findings (Table 2).

#### Forms of evidence

Participants acknowledged their need for forms of evidence to include policy briefs, media reports, newsletters, videos, success stories, testimonials, knowledge sharing and feedback mechanisms (Table 2).

### Organisational capacity for use of research evidence

#### Institutional environment to enhance use of evidence

Participants generally agreed that there was a need for ethical/regulatory committees/guidelines for research. Also highlighted was the need for proper documentation of research, which includes data inventory, improved monitoring and evaluation, establishment of a well-managed library with focal staff who circulate research/reports, and enacting laws and regulating on the utilisation of research as well as political commitment (Table 3).

**Table 3:** Participants’ response on organisational capacity needs for support of the use of evidence in policymaking relevant to maternal, newborn and child health

Institutional environment	Platforms or mechanisms	Opportunity of use of evidence	Support needs
(1) Ethical/regulatory guidelines (2) Proper documentation of research, including data inventory (3) Improved monitoring and evaluation (4) Having a stakeholder forum for dissemination of research findings (5) Improved funding for policy-relevant research (6) Political will for implementation of National Health Act, national health policy and national health research policy (7) Having a resource centre or well-managed library with focal staff, who circulate research/reports and evidence to staff (8) Enact laws/regulations on the utilisation of research evidence	(1) Website for storing and giving access to research works (2) Provision of a national database (3) Joint appointment between academia (research institutions) and health ministry (e.g. sabbaticals/secondment) (4) Functional health management information system/database (5) Accountability mechanisms at state and national levels (6) Provision of access to internet connectivity and online library (7) Availability of platforms for interactions between policymakers and research institutions regularly (8) Use of policy briefs, factsheets, etc.	(1) Policy change (2) Proposal writing and report writing (3) Monitoring and evaluation of activities (4) Making use of best practice and evaluation of their use to provide feedback and raise further research questions (5) Planning of programmes and implementation of activities (6) Decision-making and policy development	(1) Effective communication plan/strategy to disseminate research/evidence (including professional bodies) (2) Networking and collaboration (3) Physical infrastructure –conducive working environment (4) Funding and availability of evidence-based tools and resources to implement (5) Financial resources to carry out research, etc. (6) Human resources –technical support, statistical skills (7) Information communication technology (8) Logistics such as means of transportation, etc. (9) Opportunities to attend conferences and seminars

#### Platforms or mechanisms to facilitate use of evidence

Participants noted the need for the establishment of mechanisms for permanent networking and collaboration between the researchers and policymakers. Also identified as needs were a functional health management information system (HMIS)/database, internet connectivity and an online library (Table 3).

#### Opportunity of use of evidence

The key areas of opportunity to use research evidence for policy change identified by participants included report writing, proposal writing, planning of programmes, decision-making, policy development, implementation of activities, and monitoring and evaluation of activities (Table 3).

#### Support needs to use of evidence

Participants identified the financial resources to carry out research, human resources (including technical support, statistical skills), ICT, effective communication plan/strategy to disseminate research/evidence, political commitment, networking and collaboration, a physical infrastructure that will create conducive working environment, and opportunities to attend conferences, as the key areas of support they needed in order to use research evidence (Table 3).

#### Organisational initiatives relevant to evidence-informed policymaking

The responses of the stakeholders regarding organisational initiatives (mechanisms, processes, tools, strategies and platforms) relevant to evidence-informed policymaking are presented in Table 4. The stakeholders’ responses showed that the main mechanisms for use of evidence involve strategic knowledge management, operation of the department of planning, research and statistics, institutional review board, and external stakeholders/review feedback mechanisms. The main processes of evidence use included engagement of relevant stakeholders, involvement of key players in some cases, identification of problems, formation of research intervention, dissemination results, and regular tracking of programme indicators. The main tools of evidence use included forms for use in data collection routine data, research data, workers’ guidelines, HMIS, research and evaluation briefs, and score cards. The main strategies for evidence use included evaluation of results of data submitted, meeting with stakeholders, capacity building, consultative meetings, and evidence-based advocacy. The main platforms included research division of strategic knowledge management department, use of task force committee, stakeholders’ forum, e-learning platform for research skills, data repositories, research utilisation department, and internal archival systems (best practice gateway and share point programme/management meeting) (Table 4).

**Table 4:** Summary of participants’ response on the existence of organisational initiatives (mechanisms, processes, tools, strategies and platforms) relevant to evidence-informed policymaking in maternal, newborn and child health (MNCH)

Mechanisms	Processes	Tools	Strategies	Platforms
(1) Data collection of MNCH (2) Strategic knowledge management (3) Department of Planning, Research and Statistics (4) Stakeholders meeting to discuss issues (5) Institutional review board (6) External stakeholders/review feedback mechanism (7) Annual conferences	(1) Engagement of relevant stakeholders (2) Identification of problem, formation of research intervention, dissemination results (3) Process for evaluating the research proposal (4) Research priorities defined (5) Regular tracking of programme indicators	(1) Forms for use in data collection (2) Workers guideline (3) Health management information service (4) Various data tools adapted for the Federal Ministry of Health (5) Research and evaluation briefs (6) Score cards (7) Association journals	(1) Evaluation of results of data submitted (2) Training and capacity building (3) Consultative meeting with stakeholders (4) E-library to access international journals (5) Internal dissemination of evidence to programme (6) Evidence-based advocacy	(1) Research division of strategic knowledge management department (2) Management meetings (3) Using task force committee (4) Meetings of core technical committee for MNCH (5) Stakeholders forum (6) E-learning platforms and data repositories (7) Research utilisation department (8) Internal archival systems

#### Key barriers to evidence use

According to the respondents, the key barriers to evidence use in policymaking related to MNCH included no systematic way/mechanism for use of research in MNCH intervention, inadequate capacity of the organisation to conduct research that may lead to use, limited or inadequate budgetary allocation for research, no written policy that mandates staff to base their work on evidence, policymakers not interested in evidence-based facts even when research results are available, research is subject to donor rules, research priorities are donor-driven, poor dissemination of documented research results and evidence, and no interaction forum between the researchers and policymakers (Table 5).

**Table 5:** Summary of participants’ response on the barriers and facilitators of use of research evidence in policymaking related to maternal, newborn and child health (MNCH)

Key barriers to use of research evidence	Key facilitators of use of research evidence
- No systematic way/mechanism of use of research in MNCH intervention - Inadequate capacity of the organisation to conduct research that may lead to use - Limited or inadequate funding/budgetary allocation for research - Some output of research may not be of much use in programmes as they may not answer the specific questions we have - No written policy that mandates staff to base memo, proposal, etc. on evidence - Inadequate facilities for implementation - Inadequate capacity of the policymakers - Corruption and political interference - Poor political will - Weak linkages with researcher and policymaker - Lack of capacity building of policymaker on importance of research - Poor dissemination of documented research results and evidence - Non-involvement of policymakers from the beginning of research - Non-communication of research outcomes to policymaker - No interaction forum between the researchers and policymakers - Research is subject to donor rules - Research priorities are donor-driven	- Capacity building of policymakers on use of research in policy formulation - Making a policy that will make a provision of budget for research - Making a policy that will make the use of evidence in policymaking mandatory - Affiliation with an academic institution in area of generation of evidence, its interpretation and utilisation - More training/stakeholder meetings involving policymakers - Incorporate policymakers when planning and developing research - Appropriate dissemination of the research findings to relevant stakeholders - Policy should be instituted to ensure every implementation of government projects must be evidence based - Strengthening of the departments of Health Planning, Research and Strategies to play effective role in promoting research work and use - Institution of an annual forum for presentation and consideration of research work/results for possible adoption and incorporation into policymaking process - Creating a forum between the academics and policymakers - Allowing the policymakers needs to drive research projects

#### Key facilitators of evidence use

The key facilitators of use of research evidence for policymaking in MNCH identified included capacity building on use of research in policy formulation, policies to ensure appropriate budget allocations for research, policies to ensure that any newly introduced policy must be accompanied with evidence, appropriate dissemination of research findings to relevant stakeholders, use of policy briefs involving policymakers in research, allowing policymakers to determine areas of research based on needs, and establishment of an annual forum for presentation and consideration of research work/results for possible adoption and incorporation into policymaking process (Table 5).

## Discussion

This study has identified the perceived needs of policymakers and other stakeholders involved in MNCH policymaking in Nigeria as well as the barriers and facilitators to evidence-informed policymaking. To the best of our knowledge, this was the first time national MNCH policymakers, researchers and other stakeholders were brought together in Nigeria to consider issues around the research-to-policy interface and to identify areas of priority needs to support the use of research evidence in policymaking. In a 2009 report, Lavis et al. [44] noted that there is growing interest in the identification of mechanisms that enhance interactive knowledge sharing that enable research evidence to be brought together with the views, experiences and tacit knowledge of those who will be involved in, or affected by, future decisions about high-priority issues. They cited two reports by Lavis [45] and Lomas [46], which indicated that interest in identifying interactive knowledge-sharing mechanisms has been fuelled by the recognition of the need for locally contextualised ‘decision support’ for policymakers and other stakeholders to enhance the evidence-to-policy process.

Vital areas of evidence-to-policy needs of MNCH policymakers in Nigeria have been identified by this study, and this information will aid in the development of strategies to address these needs. Findings from previous studies have clearly showed that the optimal use of research evidence is not possible without taking into consideration the needs, concerns, and degree of receptivity of the potential users of this knowledge [47, 48]. In this study, participants emphasised the use of capacity building to enhance their skills for evidence-informed policymaking, creating enabling environments to support evidence, and incentives/reward to encourage stakeholders to promote use of evidence. It is well established that skills in using evidence may be improved through training and development programmes for policymakers and other policy agents. Most of the policymakers in this study identified the lack of training as their major capacity constraint.

Evidence-based skills training is very important and educating administrative officials who can then introduce new decision-making approaches to their agency is an important way to effect systemic change [49]. The importance of capacity development among policymakers and other stakeholders in the Nigerian health sector cannot therefore be overstated. This is a major factor that has the potential to boost the interest in the transfer and uptake of research evidence into policy and practice. This will positively influence governance and leadership, resources (human, material and financial), communication and quality of research [22, 23, 29, 50]. It is already a well-established fact that skills training could help policymakers and their aides not only identify research evidence that has policy relevance, but also distinguish research of high and low methodological quality [51–53].

Among the most important skills needed by the participants to enhance their use of research evidence for policymaking are skills in ICT, research methodology, scientific writing and data analysis. Findings from previous investigations have shown that a relationship exists between effective job performance of a health sector stakeholder (policymaker or service provider) and ICT use [54–56]. Dzenowagis [55] observed that ICTs have greatly improved access to health information and research, thereby supporting the health research enterprise and enabling comprehensive, evidence-based policymaking. Several studies in Nigeria have shown that policymakers’ lack of adequate capacity for ICT use constitutes a major impediment to the uptake of research evidence into the policymaking process [57–59]. Peizer [60] strongly recommended that significant time and resource commitments should be invested in training to enhance ICT competence of those involved in making health policy. This is because ICT competency will enhance the capacity for scientific writing, data analysis/management and identification of research evidence.

Participants in this study noted that the evidence-to-policy process can be given a boost in Nigeria by establishing mechanisms for permanent networking and collaboration between researchers and policymakers. Such a partnership between researchers and policymakers has been described as a crucial element for promoting the use of health research for policy development in other contexts [61, 62]. This partnership requires greater attention and consideration in developing countries, including Nigeria, where its potential utility has not been fully evaluated. Hyder et al. [63] observed that the process of translating research outcomes into policies is a critical and yet under-studied process in most developing countries. They further noted that both informal and formal mechanisms used for such translation, and the types of people involved, especially in entities like health policy units, all merit consideration [63].

In a previous study conducted in Nigeria [29], policymakers noted that, to promote the evidence-to-policy process, policymakers should be involved in the planning and execution of health research and researchers should be involved in the planning and execution of health programmes. In addition, dialogue between researchers and policymakers should be promoted, especially using a common fora or meetings, and methodologies applicable to health research should be simplified for easy understanding of policymakers.

A very important area of need the participants highlighted was the format and mechanism of communication and dissemination of research evidence, which they noted must be tailored to suit the needs of policymakers. Reference was made to the possible use of policy briefs as an example of knowledge sharing tools that can encourage policymakers to receive research evidence and use it. In a recent study on information-packaging efforts to support evidence-informed policymaking in LMICs, Adam et al. [2] observed that the importance of developing concise materials and tools to communicate various types of information to policymakers is increasingly gaining recognition. According to them, it is this recognition that has led to the development of a plethora of information-packaging efforts, which aim to support evidence-to-policy-to-practice processes based on the messages informed by research findings [5, 64, 65]. Lavis et al. [44] have indicated that policy briefs are among the most ideal type of policy information packaging tool and a new approach to improving the policymaking process by supporting evidence-informed policymaking. Some recent reports from Nigeria have shown that the use of policy briefs to promote evidence-informed policymaking is well received by policymakers [24, 66], and this is because it makes it easier for them and other stakeholders to determine whether and how the available research evidence accords with their own beliefs, values, interests or political goals and strategies [44].

It is noteworthy that the participants emphasised their need for political commitment from the government and financial resources to support evidence-to-policy processes as well as physical infrastructure and conducive working environments. These needs are supported by several reports, which clearly showed that there is a connection between political interest, funding and infrastructure to promote the uptake of research into policy [31, 67, 68]. According to Deans and Ademokun [67], apart from capacity to use research to achieve evidence-informed policymaking, many other factors, including political will and funding constraints, affect the likelihood of policy being informed by evidence. In a study that explored barriers to research utilisation in policy formulation in Egypt [30], the availability of funds to support the implementation of the findings (90.7%) and political interests (81.3%) ranked as the most important factors affecting the policymaking process. Furthermore, El-Jardali et al. [31] noted that 79.2% of their study respondents reported that limited public funding for the health sector and the values of the governing parties (53.9%) exerted a strong influence on the policymaking process. Evidence from previous studies has demonstrated that improving research infrastructure, political commitment and financial resources towards the evidence-to-policy process can provide an enabling environment for the use of research evidence for policymaking and practice [61, 69].

Another noteworthy observation by the participants is the need for researchers to spend their sabbatical leave in government ministries, departments and agencies to increase interaction between policymakers and researchers. Conversely, policymakers should be encouraged to spend some time in research institutions. This type of staff exchange strategy, or secondment programme, has the potential to enhance better interaction between policymakers and researchers.

Secondments have been shown to offer the opportunity to enhance personal development and working practices for front-line staff through valuable first-hand encounters, whereby the secondee will experience new concepts, values and cultures that can test their ability to succeed in a different environment [6]. Secondments can therefore provide a positive way of motivating people and increasing work satisfaction, whilst enhancing best practice, collaborative working partnerships, knowledge and skills [70]. Although the practice of secondment is generally known as a strategy for skills development for mutual organisational and individual benefit, there is, however, a gap in the literature regarding practical implementation considerations and critical success factors related to the use of secondment as a global strategy to promote evidence-informed health policy development and implementation strategy [71]. This merits further consideration in future studies.

It is interesting to note that the respondents acknowledged the existence of some organisational initiatives, including mechanisms, processes, tools, strategies and platforms, which have the potential to enhance the use of research evidence in policymaking. Some previous reports have portrayed the existence of similar organisational initiatives designed to promote the use of research evidence in policymaking [32, 48, 72]. An important example is the Nigeria Evidence-Based Health System Initiative, which was established with the aim of building a responsive evidence-based health system, with emphasis on primary healthcare, to improve MNCH outcomes [73–76]. The FMOH and all the 36 state ministries of health in Nigeria have two important units known as the Department of Planning, Research and Statistics and the HMIS division. The policymakers in this study admitted that these units are among the main initiatives designed to promote evidence-informed policymaking because the units are involved in the (1) identification of problems and formation of research intervention; (2) dissemination of results and regular tracking of programme indicators; (3) data repositories and internal archival systems; (4) strategic knowledge management and research utilisation; and (5) programme monitoring/evaluation and performance assessment.

However, it is pertinent to state that the existence of these organisational initiatives, which may be regarded as incentives or motivations to use research, does not guarantee their effective engagement and utilisation by policymakers. A major factor that may be responsible for inadequate engagement of these organisational initiatives could be the lack of basic research capacity and competence among policymakers. This is an important factor identified as a major barrier to the use of research evidence in policymaking in this study, and which appears to be a common denominator across many LMICs [9, 10, 22, 32]. An earlier study observed that the demand for research evidence is not only influenced by policymakers’ incentives or motivations to use research, but more importantly by their capacity to access, understand and use research [77].

In addition to inadequate research capacity, other key barriers to the use of research evidence in policymaking identified by the participants in this study are consistent with the report of several other studies that assessed policymakers’ perceived barriers to use of evidence in policymaking [32, 62, 78–80]. Undoubtedly, the barriers identified in this study may be playing a major contributory role in the failure to develop effective evidence-informed health policy related to MNCH in Nigeria. They could also be responsible for the lack of adequate engagement and utilisation of numerous organisational initiatives instituted to promote the use of research evidence in the policymaking process in Nigeria. According to Davis and Davis [81], and Ellen et al. [82], these barriers are not easily overcome, but learning opportunities and their assessment are potential mechanisms that could be useful steps towards developing opportunities to address them.

Interestingly, all the main facilitators of use of research evidence in policymaking identified by policymakers in this study are consistent with the reports from numerous previous studies [32, 62, 80]. The policymakers were emphatic about the need for capacity building on use of research in policy formulation, appropriate dissemination of the research findings to relevant stakeholders, and involvement of policymakers in research. Policymakers in several studies have consistently suggested that implementation of these facilitators can enhance the uptake of research evidence into the policymaking process [78–81].

### Study limitations

This study had limitations. First, the self-assessment method we used is known to be subject to self-esteem bias, may be unreliable and is difficult to validate [83]. Highlighting the weakness of this technique, Deans and Ademokun [67] noted that being able to critically recognise and understand one’s own gaps in skills and knowledge is a difficult process that takes guided thought. The second limitation was our inability to employ interactional content analysis of the group consultation outcome. This is advocated in future studies. It is acknowledged that these limitations may have reduced the amount and depth of valuable information that could have been generated from this study; however, we believe that the quality of data was not adversely affected. The findings can serve as reliable first step towards the development of effective interventional strategy to improve the MNCH evidence-to-policy process in Nigeria.

## Conclusion

The information provided by the respondents in this study has revealed the areas of needs as well as the barriers and facilitators to use of research evidence in policymaking in Nigeria. This information is highly valuable in the development of specific intervention strategies at individual and organisational levels that will facilitate the evidence-to-policy process. To enhance the evidence-informed policymaking process, it is important to improve organisational initiatives that promote the use of research evidence, for example, research commissioning by policymaking organisations. It is also important to improve research infrastructure, funding and training of policymakers, and to establish sustainable platforms for policymakers and researchers’ interaction. Both individual and institutional strengthening interventions have the potential to promote evidence-informed policymaking, although studies assessing the impact of these on evidence-to-policy processes are essentially lacking. Future studies assessing the impact of interventions designed to address the barriers associated with evidence uptake in the policymaking process are advocated.

## Additional file

Additional file 1: Improving maternal and child health policymaking processes in Nigeria. (DOCX 13 kb)

## Abbreviations

FMOH: Federal Ministry of Health
HMIS: health management information service
ICT: information and communication technology
LMICs: low- and middle-income countries
MDGs: Millennium Development Goals
MNCH: maternal, newborn and child health
WAHO: West African Health Organisation

## References

[1] World Health Assembly (2005). World Health Assembly Concludes: Adopts Key Resolutions Affecting Global Public Health.

[2] Adam T, Moat KA, Ghaffar A, Lavis JN (2014). Towards a better understanding of the nomenclature used in information-packaging efforts to support evidence-informed policymaking in low- and middle-income countries. Implement Sci.

[3] Panisset U, Koehlmoos TP, Alkhatib AH, Pantoja T, Singh P, Kengey-Kayondo J, McCutchen B (2012). Implementation research evidence uptake and use for policy-making. Health Res Policy Syst.

[4] Koon AD, Rao KD, Tran NT, Ghaffar A (2013). Embedding health policy and systems research into decision-making processes in low- and middle-income countries. Health Res Policy Syst.

[5] Rosenbaum SE, Glenton C, Wiysonge CS, Abalos E, Mignini L, Young T, Althabe F, Ciapponi A, Marti SG, Meng Q, Wang J, la Hoz Bradford AM, Kiwanuka SN, Rutebemberwa E, Pariyo GW, Flottorp S, Oxman AD (2011). Evidence summaries tailored to health policy-makers in low- and middle-income countries. Bull World Health Organ.

[6] Oxman AD, Bjørndal A, Becerra-Posada F, Gibson M, Block MA, Haines A, Hamid M, Odom CH, Lei H, Levin B, Lipsey MW, Littell JH, Mshinda H, Ongolo-Zogo P, Pang T, Sewankambo N, Songane F, Soydan H, Torgerson C, Weisburd D, Whitworth J, Wibulpolprasert S (2010). A framework for mandatory impact evaluation to ensure well informed public policy decisions. Lancet.

[7] World Health Organization (2004). World Report on Knowledge for Better Health-Strengthening Health Systems.

[8] Alliance for Health Policy and Systems Research (2007). Briefing Note Number 1: What is Health Policy and Systems Research and why does it matter?.

[9] González-Block MA, Mills A (2003). Assessing capacity for health policy and systems research in low and middle income countries. Health Res Policy Syst.

[10] Shroff Z, Aulakh B, Gilson L, Agyepong IA, El-Jardali F, Ghaffar A (2015). Incorporating research evidence into decision-making processes: researcher and decision-maker perceptions from five low- and middle-income countries. Health Res Policy Syst.

[11] Holmes BJ, Schellenberg M, Schell K, Scarrow G (2014). How funding agencies can support research use in healthcare: an online province-wide survey to determine knowledge translation training needs. Implement Sci.

[12] Oxman AD, Lavis JN, Lewin S, Fretheim A (2009). SUPPORT Tools for evidence-informed health Policymaking (STP) 1: What is evidence-informed policymaking?. Health Res Policy Syst.

[13] World Health Organization (2000). The World health report 2000: health systems: improving performance.

[14] United States Agency for International Development (2008). Working Toward the Goal of Reducing Maternal and Child Mortality: USAID Programming and Response.

[15] Galandanci H, Ejembi C, Iliyasu Z, Alagh B, Umar U (2007). Maternal health in Northern Nigeria – a far cry from ideal. BJOG.

[16] Federal Ministry of Health (2007). Integrated Maternal, Newborn and Child Health Strategy.

[17] Ebonyi State Mother and Child Care Initiative (MCCI) Nigeria (2010). Documentation commissioned by The United Nations Population Fund (UNFPA).

[18] National Population Commission Nigeria and ICF International (2014). Nigeria Demographic and Health Survey 2013.

[19] National Population Commission Nigeria (2009). Nigeria Demographic and Health Survey 2008.

[20] National Population Commission and ORC Macro (2004). Nigeria Demographic and Health Survey 2003.

[21] World Bank. Data – Mortality Rate Under 5 (per 1,000 live births). The World Bank Group 2015. http://data.worldbank.org/indicator/SH.DYN.MORT. Accessed 4 June 2016.

[22] Uneke CJ, Ezeoha AE, Ndukwe CD, Oyibo PG, Onwe F, Igbinedion EB, Chukwu PN (2011). Individual and organisational capacity for evidence use in policy making in Nigeria: an exploratory study of the perceptions of Nigeria health policy makers. Evidence Policy.

[23] Uneke CJ, Ezeoha AE, Ndukwe CD, Oyibo PG, Onwe F (2012). Promotion of evidence-informed health policymaking in Nigeria: bridging the gap between researchers and policymakers. Global Public Health.

[24] Onwujekwe O, Uguru N, Russo G, Etiaba E, Mbachu C, Mirzoev T, Uzochukwu B (2015). Role and use of evidence in policymaking: an analysis of case studies from the health sector in Nigeria. Health Res Policy Syst.

[25] Federal Ministry of Health (2015). Nigeria’s Call to Action to Save Newborn Lives.

[26] World Bank (2015). Saving One Million Lives Initiative Nigeria.

[27] Federal Ministry of Health (2010). National Guidelines for the Prevention of Mother to Child Transmission of HIV (PMTCT).

[28] Partnership for Reviving Routine Immunization in Northern Nigeria – Maternal, Newborn & Child Health (PRRINN-MNCH) Initiative (2010). Kangaroo Training Guidelines for Low Birth Weight Babies.

[29] Uneke CJ, Ezeoha A, Ndukwe CD, Oyibo PG, Onwe F (2010). Development of health policy and systems research in Nigeria: lessons for developing countries’ evidence-based health policy making process and practice. Healthc Policy.

[30] Abou-Zeid A, Galal Y, Shawky M, El-Rabbat M (2012). Exploring barriers to research utilization in policy formulation in Egypt: researchers’ perspectives. J Am Sci.

[31] El-Jardali F, Lavis JN, Ataya N, Jamal D, Ammar W, Raouf S (2012). Use of health systems evidence by policymakers in eastern Mediterranean countries: views, practices, and contextual influences. BMC Health Serv Res.

[32] Oliver K, Innvar S, Lorenc T, Woodman J, Thomas J (2014). A systematic review of barriers to and facilitators of the use of evidence by policymakers. BMC Health Serv Res.

[33] Graham ID, Logan J, Harrison MB, Straus SE, Tetroe J, Caswell W, Robinson N (2006). Lost in knowledge translation: time for a map?. J Contin Educ Health Prof.

[34] Graham ID, Tetroe J (2007). Some theoretical underpinnings of knowledge translation. Acad Emerg Med.

[35] Lavis JN (2006). Research, public policymaking, and knowledge translation processes: Canadian efforts to build bridges. J Contin Educ Health Prof.

[36] World Health Organization (2012). Asia-Pacific Leadership and Policy Dialogue for Women’s and Children’s Health.

[37] Mirzoev T, Green A, Gerein N, Pearson S, Bird P, Ha BTT, Ramani K, Qian X, Mukhopadhyay M, Soors W (2013). Role of evidence in maternal health policy processes in Vietnam, India and China: findings from the HEPVIC project. Evidence Policy.

[38] Bowen S, Zwi AB (2005). Pathways to “evidence-informed” policy and practice: a framework for action. PLoS Med.

[39] Dobrow MJ, Goel V, Upshur REG (2004). Evidence-based health policy: context and utilisation. Soc Sci Med.

[40] Hanney SR, Gonzalez-Block MA, Buxton MJ, Kogan M (2003). The utilisation of health research in policy-making: concepts, examples and methods of assessment. Health Res Policy Systems.

[41] Canadian Health Services Research Foundation (CHSRF) (2008). Validating the Foundation’s Self-assessment Tool: A Summary.

[42] Giorgi A, Giorgi A (1985). Sketch of a Psychological Phenomenological Method. Phenomenology and Psychological Research: Essays.

[43] Albert MA, Fretheim A, Maïga D (2007). Factors influencing the utilization of research findings by health policy-makers in a developing country: the selection of Mali’s essential medicines. Health Res Policy Syst.

[44] Lavis JN, Permanand G, Oxman AD, Lewin S, Fretheim A (2009). SUPPORT Tools for evidence-in-formed health policymaking (STP) 13: preparing and using policy briefs to support evidence-in-formed policymaking. Health Res Policy Syst.

[45] Lavis JN (2006). Moving forward on both systematic re-views and deliberative processes. Healthc Policy.

[46] Lomas J (2007). Decision support: a new approach to making the best healthcare management and policy choices. Healthc Q.

[47] Berman J, Mitambo C, Matanje-Mwagomba B, Khan S, Kachimanga C, Wroe E, Mwape L, van Oosterhout JJ, Chindebvu G, van Schoor V, Puchalski Ritchie LM, Panisset U, Kathyola D (2015). Building a knowledge translation platform in Malawi to support evidence-informed health policy. Health Res Policy Syst..

[48] Dagenais C (2010). Knowledge transfer in community-based organizations: A needs assessment study. Global J Commun Psychol Pract.

[49] Green A, Bennett S (2007). Sound Choices: Enhancing Capacity for Evidence-Informed Health Policy.

[50] Uneke CJ, Ezeoha AE, Ndukwe CD, Oyibo PG, Onwe F (2012). Enhancing Leadership and Governance Competencies to Strengthen Health Systems in Nigeria: Assessment of Organizational Human Resources Development. Healthc Policy.

[51] Uneke CJ, Ezeoha AE, Uro-Chukwu H, Ezeonu CT, Ogbu O, Onwe F, Edoga C (2015). Enhancing health policymakers’ information literacy knowledge and skill for policymaking on control of infectious diseases of poverty in Nigeria. Online J Public Health Inform.

[52] Uneke CJ, Ezeoha AE, Uro-Chukwu H, Ezeonu CT, Ogbu O, Onwe F, Edoga C (2015). Improving Nigerian health policymakers’ capacity to access and utilize policy relevant evidence: outcome of information and communication technology training workshop. Pan Afr Med J.

[53] Uneke CJ, Ezeoha AE, Uro-Chukwu H, Ezeonu CT, Ogbu O, Onwe F, Edoga C (2015). Enhancing the capacity of policy-makers to develop evidence informed policy brief on infectious diseases of poverty in Nigeria. Int J Health Policy Manag.

[54] Chetley A, Davies J, Trude B, McConnell H, Ramirez R, Shields T, Drury P, Kumekawa J, Louw J, Fereday G, Nyamai-Kisia C. Improving Health, Connecting People: The Role of ICTs in the Health Sector of Developing Countries. A Framework Paper: Information for Development Program (Infodev) Working paper No. 7. London: Worldlink Worldwide; 2006.

[55] Dzenowagis J (2009). Bridging the Digital Divide: Linking Health and ICT Policy.

[56] United Nations Economic and Social Council. Economic Commission for Africa (2001-08). Information and Communication Technology for Health Sector: The African Development Forum ‘99 Post ADF Summit. UN. ECA Committee on Development Information Meeting, September 4–7, Addis Ababa; 2001. http://repository.uneca.org/handle/10855/506. Accessed 4 June 2017.

[57] Alabi GA (1994). Case Study Effectiveness of Informatics Policy Instruments in Africa: Nigeria.

[58] Akande JO, Jegede PO (2004). Andragogy and computer literacy: the Nigerian perspective. African Symposium.

[59] Uneke CJ, Ezeoha AE, Ndukwe CD, Oyibo PG, Onwe F (2011). Enhancing health policymakers’ capacity to use information and communication technology in Nigeria. J Health Inform Dev Countr.

[60] Peizer J. Bridging the Digital Divide: First you Need the Bridge. 2000. https://www.socialtext.net/m/page/ourmedia/bridging_the_digital_divide. Accessed 4 June 2016.

[61] Campbell DM, Redman S, Jorm L, Cooke M, Zwi AB, Rychetnik L (2009). Increasing the use of evidence in health policy: practice and views of policy makers and researchers. Aust New Zealand Health Policy.

[62] Innvær S, Vist G, Trommald M, Oxman A (2002). Health policy-makers perceptions of their use of evidence: a systematic review. J Health Serv Res Policy.

[63] Hyder AA, Bloom G, Leach M, Syed SB, Peters DH (2007). Future health systems: innovations for equity. Exploring health systems research and its influence on policy processes in low income countries. BMC Public Health..

[64] Lavis JN (2009). How can we support the use of systematic reviews in policymaking?. PLoS Med.

[65] Lavis JN, Lomas J, Harnid M, Sewankambo NK (2006). Assessing country-level efforts to link research to action. Bull World Health Organ.

[66] Uneke CJ, Ezeoha AE, Uro-Chukwu H, Ezeonu CT, Ogbu O, Onwe F, Edoga C (2015). Promoting evidence to policy link on the control of infectious diseases of poverty in Nigeria: outcome of a multi-stakeholders policy dialogue. Health Promot Perspect.

[67] Deans F, Ademokun A (2004). Investigating Capacity to Use Evidence.

[68] Mackenbach JP, McKee M (2015). Government, politics and health policy: A quantitative analysis of 30 European countries. Health Policy.

[69] Santesso N, Tugwell P (2006). Knowledge translation in developing countries. J Contin Educ Health Prof.

[70] MWH UK. Understanding Secondments in MWH. 2013. http://docs.healthandsafetyhub.co.uk/MWH/Training_materials/understanding-secondments.pdf. Accessed 4 June 2016.

[71] Grignon JS, Ledikwe JH, Makati D, Nyangah R, Sento BW, Semo B (2014). Maximizing the benefit of health workforce secondment in Botswana: an approach for strengthening health systems in resource-limited settings. Risk Manag Healthc Policy.

[72] Jewell CJ, Bero LA (2008). “Developing good taste in evidence”: facilitators of and hindrances to evidence-informed health policymaking in state government. Milbank Q.

[73] Nigeria Evidence-based Health System Initiative (NEHSI). Overview of the Project. 2007. https://www.idrc.ca/sites/default/files/sp/Documents%20EN/NEHSI-Update-English.pdf. Accessed 21 May 2016.

[74] CIET Video on NEHSI Social Audit – CIET Methods Documentary. 2013. https://www.youtube.com/watch?v=0CyYa8eJNww. Accessed 4 June 2016.

[75] NEHSI Docudrama on Childhood Illnesses, Bauchi State, Nigeria. 2012. https://www.youtube.com/watch?v=ahHpwqhndg8. Accessed 4 June 2016.

[76] Andersson N (2011). Proof of impact and pipeline planning: directions and challenges for social audit in the health sector. BMC Health Serv Res.

[77] Newman K, Fisher C, Shaxson L (2012). Stimulating demand for research evidence: what role for capacity building?. IDS Bull.

[78] El-Jardali F, Lavis JN, Ataya N, Jamal D (2012). Use of health systems and policy research evidence in the health policymaking in eastern Mediterranean countries: views and practices of researchers. Implement Sci..

[79] Corluka A, Hyder AA, Segura E, Winch P, McLean RKD (2015). Survey of Argentine health researchers on the use of evidence in policymaking. PLoS One.

[80] Orton L, Lloyd-Williams F, Taylor-Robinson D, O'Flaherty M, Capewell S (2011). The use of research evidence in public health decision making processes: systematic review. PLoS One.

[81] Davis D, Davis N, Straus S, Tetroe J, Graham G (2009). Educational interventions. Knowledge Translation in Healthcare: Moving from Evidence to Practice.

[82] Ellen ME, Léon G, Bouchard G, Lavis JN, Ouimet M, Grimshaw JM (2013). What supports do health system organizations have in place to facilitate evidence-informed decision-making? A qualitative study. Implement Sci.

[83] Haahr JH, Shapiro H, Sørensen S (2004). Defining a Strategy for the Direct Assessment of Skills.

