# Supplementary material for: Improving maternal and child health policymaking processes in Nigeria: an assessment of policymakers’ needs, barriers and facilitators of evidence-informed policymaking
Source: Health Res Policy Syst. 2017 Jul 12;15(Suppl 1):48. doi: 10.1186/s12961-017-0217-5 (PMC5516839; doi:10.1186/s12961-017-0217-5)
Supplement: Additional file 1: — Improving maternal and child health policymaking processes in Nigeria. (DOCX 13 kb) [file 12961_2017_217_MOESM1_ESM.docx]

Additional File Improving maternal and child health policymaking processes in Nigeria

**Assessment of policymakers’ needs, barriers and facilitators of evidence-informed policymaking**

**1**. **Individual capacity for use of research evidence**

***(i). Aptitudes:*** to strengthen your aptitudes for use evidence, what are the intervention that are important for you?

***(ii). Skills***: what types of skills do you need to better use evidence and research findings?

***(iii). The sources of evidence***: which sources of evidence or research results would you like to access to improve your use of evidence and research findings?

***(iv). Forms of evidence:*** In what forms would you like to receive evidence and research findings to help you to use them?

**2. Organisational capacity for use of research evidence**

***(i). Institutional environment***: what types of improvement (laws, regulations, service organization, support, motivation) do you think are important in your workplace or your country that can help you to better use evidence and research findings?

***(ii). Platforms or mechanisms***:

a) What types of platforms or mechanisms do you think are important to put in place or reinforce in your workplace or your country for facilitate your access to evidence and research findings?

b) What types of platforms or mechanisms do you think important to put in place or reinforce in your workplace or your country to facilitate your every time use of evidence and research findings?

***(iii). Opportunity of use of evidence*:** what activities in your daily work are opportunities for you, to permanent use of evidence and research findings?

***(iv). Support needs***: what types of support would you like to receive when you decide to use evidence and research findings?

**3. Organisational initiatives relevant to evidence-informed policymaking**

Briefly describe organizational initiatives relevant to evidence-informed policymaking under the following terms:

(i). Mechanisms

(ii). Processes

(iii). Tools

(iv). Strategies

(v). Platforms

**4. Barriers and facilitators of use of evidence in policymaking**

(i). Key barriers to use of research evidence

(ii). Key facilitators of use of research evidence
